# No Time to Read

**Published:** 1906-09

**Authors:** 


### NO TIME TO READ.

Once in a while we get a letter from some doctor ordering the CLINIC discontinued "because I have no time to read." This always makes me smile. I like to see a doctor busy, for to be busy means (or should) plenty of money in the purse and some in the bank—a well-stocked medicine cabinet, more and better surgical instruments, an office furnished with all the contraptions which help in business boosting, shelves full of books, and tables piled with medical magazines. That's real prosperity. These things mean that here is a doctor who is so much alive to his own interests, as well as to those of his patients, that he is going to have all of the tools of his trade about him so he can constantly improve the quality of his work—do as well as the next fellow and command as big fees.

There is something radically wrong with the man who has "no time to read." If he hasn't the time he should take the time, just as he should to eat and sleep. How else can he know what is going on in the medical world? What advances are being made? Does it never occur to him that the reason he lost that case yesterday was because he is *already* behind the times—even though he is out of college less than five years? The fact that very likely would have saved the life was in the magazine (likely in the CLINIC) which he never took the trouble to open. No matter how successful he may be, sooner or later he will be replaced in the affections and confidence of the community by young Jones who has hard scrabbling enough now, God knows, but who is forging to the front, because *he* has "the time to read."

It's a strange thing, but you never hear of any men of the first eminence in the profession who have no time to read. Yet they must be busy or all signs fail, for how else did they attain their eminence except by knowing things that others did not know and doing things that others could not do. Read?

Why, these men are continually reading. In their "spare moments" they not only keep up with the profession, but keep ahead of it. The queer part of it is that these men have also time for recreation, for outside non-medical avocations. Virchow was a master in anthropology as well as in medicine, Billroth played the piano and knew music like a master, Weir Mitchell not only made a place for himself at the head of the psychiatrists but is one of the greatest of American novelists, while our own Senn, in the hours when other men sleep, writes medical books, books of travel, and even now and then breaks out in verse!

"No time to read?" My dear friend it isn't so. The trouble is that you are too blooming lazy (pardon the lapsus, a less forcible word will not do and we dare not use a stronger); you had rather take a nap or have a "quiet smoke" after the labors of the day, or spend your time in some other idle way, than to get right down to this building business—this making of better doctors. Gradually, how gradually you can hardly say, you got "out of the notion," and now you delude yourself with the belief that you are "too busy!" My poor friend, you are going to have time enough "for reading" or anything else after a bit. Really, wouldn't it be better to take a little time right now, and keep "in the swim?" "Work?" Of course it is, but it pays.—*Alk. Clinic.*

